# Rules of Brain Anatomy—Revised

**Published:** 1899-05

**Authors:** William C. Krauss

**Affiliations:** Buffalo, N. Y.


### RULES OF BRAIN ANATOMY—REVISED.

By WILLIAM C. KRAUSS, M. D., Buffalo, N. Y.

### RULE OF TWO.

- Nerve centers:
  1. Encephalon. 2. Myelon.
- Encephalon:
  1. Cerebrum. 2. Cerebellum.
- Hemispheres:
  - Cerebrum / Cerebellum / Myelon — 1. Right. 2. Left.
- Fissures:
  1. Longitudinal. 2. Transverse.
- Dura-folds:
  1. Falx. 2. Tentorium.
- Brain-matter:
  1. White. 2. Gray.

---

### RULE OF THREE.

- Membranes:

  1. Dura. 2. Arachnoid. 3. Pia.

- Hemispheral fissures:

  1. Sylvian. 2. Central (Rolandic). 3. Parieto-occipital.

- Convolutions:

  - Lobes:

    - 1. Frontal — 1. First or Super. 2. Second Medi. 3. Third Sub.

    - 2. Temporal — 1. First or Super. 2. Second Medi. 3. Third Sub.

    - 3. Occipital — 1. First or Super. 2. Second Medi. 3. Third Sub.

- Commissures:

  1. Pre. 2. Medi. 3. Post.

- Basal ganglia, pairs:

  1. Striata. 2. Thalami. 3. Quadrigemina.

- Cerebellum:

  1. Right Hemisphere. 2. Left hemisphere. 3. Vermis.

- Cerebellar peduncles, pairs:

  1. Pre. 2. Medi. 3. Post.

- Cerebellar cortex-layers:

  1. Granular. 2. Purkinje's. 3. Molecular.

- Cranial nerves, pairs:

  W-i-l-l-i-s plus 3= 9.

  S-ö-m-m-e-r-i-n-g plus 3=12.

---

### RULE OF FIVE.

- Hemispheral lobes:
  *Visible:* 1. Fiontal. 2. Parietal. 3. Temporal. 4. Occipital.
  *Invisible:* 5. Insula.
- Ventricles:
  *Visible:* 1. First. 2. Second. 3. Third. 4. Fourth.
  *Invisible:* 5. Fifth (pseudo-ventricle).
- Cerebral cortex-layers:
  1. Molecular. 2. Small pyramidal cells. 3. Large pyramidal cells. 4. Polymorphic cells. 5. White matter.

